# Immuno-dominant dengue NS1 peptides as antigens for production of monoclonal antibodies

**DOI:** 10.3389/fmolb.2022.935456

**Published:** 2022-10-21

**Authors:** Erandi Munasinghe, Maheshi Athapaththu, Wimaladharma Abeyewickreme

**Affiliations:** ^1^ Faculty of Medicine, University of Kelaniya, Ragama, Sri Lanka; ^2^ Industrial Technology Institute, Colombo, Sri Lanka; ^3^ Department of Para Clinical Sciences, Faculty of Medicine, General Sir John Kotelawala Defence University, Ratmalana, Sri Lanka

**Keywords:** dengue NS1 protein, peptide antigens, dengue detection, ELISA, monoclonal antibodies

## Abstract

Synthetic peptides have recently become common as antigens for antibody production. Peptides are short chains of amino acids that can be used to elicit an immune response. The immunogenicity of the peptide antigens varies depending on the length, charge, solubility, and amino acids contained in the peptide sequence. Dengue NS1 protein is an important target antigen in the early detection of dengue infection. In this study, peptides corresponding to a highly conserved region from the dengue NS1 region were designed and synthesized. Balb/C mice were immunized against each peptide and spleen cells extracted from the immunized mice were fused with NS0 murine myeloma cells. Hybridoma clones obtained from the fusions were tested against peptides using ELISA. Out of 1,830 growing clones, 28 clones produced antibodies reacting with dengue NS1 peptides. A purified monoclonal antibody reacting with all four peptides was tested for reactivity with dengue NS1 native protein using dengue-confirmed serum and urine samples. The monoclonal antibody shows significant reactivity with both serum and urine. The findings of the current research can be used to detect dengue infection using urine, which ultimately results in the prevention of dengue epidemics through painless diagnosis, following treatment, and patient management to safeguard human and economic wellness.

## Introduction

Dengue fever is caused by a positive-strand RNA virus from the genus flavivirus, which belongs to the family Flaviviridae (Tuiskunen Bäck and Lundkvist, 2013), which infects mammals (Thoisy et al., 2009). *Aedes aegypti* and *Aedes albopictus* mosquitoes those act as the vectors are mainly accountable for the spread of infection. In Asian countries, *A. aegypti* contributes to most of the infected population (Kanakaratne et al., 2009). The global burden of dengue infection is expanded by its genetically distinct, four virus serotypes. These four serotypes, termed dengue virus (DENV) 1, DENV 2, DENV 3, and DENV 4 (Guzman and Harris, 2015), carry more than 30% difference in the amino acid sequences of their genome (Soo et al., 2016). According to the statistics, these four serotypes are diversely spread through different regions in the world. There is an intermittent rise and fall in the serotypes, especially in countries where all four serotypes prevail. Among these, DENV 2 and 3 have accounted for the highest number of dengue cases in countries such as Indonesia (Sasmono et al., 2018), Sri Lanka (Wijewickrama et al., 2018), East Africa, and Latin Americas (Messer et al., 2003) for decades. Since the 1990s, DENV 1, 2, and 3 have intermittently circulated in Middle Eastern countries (Amarasinghe and Letson, 2012). Especially in Sri Lanka (Kanakaratne et al., 2009), Indonesia (Sasmono et al., 2018) and the other southeast Asian countries (Bhatia et al., 2013), all these four serotypes have been co-circulating eccentrically in the urban parts of the countries for over three decades.

Detecting dengue fever is accomplished by several molecular and immunological methods. The serological detection of the virus, viral proteins, or antibodies present in the blood is the most commonly used method. Virus isolation is considered the most reliable detection technique. Nevertheless, this method consumes more time than the other methods, and the sensitivity of the test largely depends on sample storage and handling since DENV particles are liable to heat (Grard et al., 2014; Gyawali and Taylor-Robinson, 2017).

Molecular diagnosis using conventional polymerase chain reaction (PCR), real-time reverse-transcriptase PCR, and nested PCR detects the dengue viral genome in acute-phase blood samples. The molecular methods for genomic detection have produced highly sensitive results, even though these require high-cost equipment and well-trained technical staff (World Health Organization, 2009).

In the serological detection of dengue infection, many antigenic proteins such as viral nonstructural (NS) proteins and envelope proteins are used. Among the several NS proteins, dengue NS1 and NS5 proteins are used as antigens and NS1 protein is the most commonly used since it is abundantly available in the patient’s blood from the onset of fever (Cuzzubbo et al., 2001; Ambrose et al., 2017). Antibody detection can be performed for IgG and IgM subclasses of immunoglobulins. IgM antibodies can be detected from 4 to 5 days of primary infection and are available for up to 3 months. IgG subclass can be detected after a week of the onset of fever and is available for years. Both antigen and antibody detection can be performed using enzyme-linked immunosorbent assay (ELISA). Despite advantages such as high sensitivity and specificity, these assays have been limited to routine laboratory diagnosis. Currently, rapid diagnosis using lateral flow immunoassay (LFIA) or strip test plays a major role in dengue detection as a result of its high efficiency (Ty Hang et al., 2009; World Health Organization, 2009).

Monoclonal antibodies play an inevitable role in the development of diagnostics (Mahmuda et al., 2017). These single-epitope antibodies have high efficiency and sensitivity in detecting viral proteins, viruses, and other antigenic substances (Gubler et al., 1984; Gerna et al., 1990). Traditionally, for the development of monoclonal antibodies, whole virus particles or recombinant full-length viral proteins are used. Phage display and DNA-based immunizations are also being used to elicit an immune response for monoclonal antibody production (Liu et al., 2002; Athmaram et al., 2013; Liu et al., 2016). The use of these substances for monoclonal antibody generation has its own downside as well as apparent advantages. The requirement of costly equipment and reagents, biosafety issues, and longer times for the synthesis and purification of proteins can be described as the so-called drawback.

Synthetic peptides have recently become common as antigens for antibody production. Peptides are short chains of amino acids that can be used to elicit an immune response. Even though these do not display the native conformation of natural proteins, a peptide can give an exact epitope resulting in low cross-reactivity as an antigen (Liew et al., 2021). The synthesis of peptides considerably demands low costs and takes shorter time , which makes researchers more interested in using peptide antigens. Peptide immunogenicity is a critical factor that causes a strong immune response and the production of desired antibodies. The immunogenicity of the peptide antigens varies depending on the length, charge, solubility, and amino acids contained in the peptide sequence (Lee et al., 2016).

DENV NS1 is a 55-kDa glycoprotein that is expressed in both membrane-associated and secreted forms. It is secreted as a soluble hexamer by DENV-infected cells since the protein is expressed on the surface of the infected cells. NS1 protein is an important target antigen in the early detection of DENV infection since this is expressed within the first 10 days of illness. Due to its high availability in the blood of infected patients, DENV NS1 protein is considered an effective antigen and a good marker for the early detection of dengue infections. The DENV NS1 protein monomer consists of three structural domains, namely, the β-roll dimerization domain, wing domain, and β-ladder domain. The NS1 hexamer crystal structure shows a barrel-shaped oligomer with a central open channel. The three dimers are arranged symmetrically in such a way that the β-roll domains are entirely facing inward and the channel interior is lined by the hydrophobic protrusion surface contributed by each dimeric component. In contrast to the β-roll domains, the glycosylation sites and most of the linear epitopes of the identified NS1 face outward, representing the most accessible parts of the NS1 hexamer to host antibodies (Yap et al., 2017). Hence, even if the proteins are glycosylated during natural infection, the monoclonal antibodies produced against the DENV NS1 antigens can react positively. Therefore, this study focuses on the development of monoclonal antibodies against synthetic peptides designed from the DENV NS1 protein.

## Methods

The reagents for this study were purchased from Sigma-Aldrich, United States, for all the experiments, unless otherwise stated.

### Designing of peptides against dengue NS1 protein

Peptides corresponding to a highly conserved region from DENV NS1 were designed and commercially synthesized from 21st Century Biochemicals, United States. The peptide sequences were selected from the work published by Masrinoul et al. (2011). Four DENV serotype-specific peptides, namely, peptide 1, peptide 2, peptide 3, and peptide 4, were redesigned considering numerous factors. The sequence of the Sri Lankan isolates, the abundance of amino acid residues that causes oxidation, solubility, length of the peptide, N-terminal and C-terminal amino acids, and multiple amino acid residues were considered when designing the peptides (Hancock and OReilly, 2005). Keyhole limpet hemocyanin (KLH) was used as the carrier protein which is attached to the peptides during synthesis.

### Production of anti-dengue NS1 monoclonal antibodies

Four female Balb/C mice were immunized subcutaneously against each peptide, mixed (1:1) with Sigma adjuvant system (Sigma S6322). In order to immunize the mice for each peptide, initially, each mouse was injected with 25 µg of the selected peptide and three booster doses were performed with 25 µg of the peptide in 2-week intervals. Three days after the final injection, the spleen of the mice was excised aseptically and the extracted spleen cells were fused with the NS0 murine myeloma cells in a 5:1 ratio using polyethylene glycol. The successfully fused cells were selected using HAT media (Gibco^®^, United Kingdom) supplemented with 20% fetal bovine serum (Hyclone™, United States) (Korbakis et al., 2015).

The cell culture supernatants (100 µL) of hybridoma clones were screened for the presence of antibodies against dengue serotype–specific peptides, as described below. The antibodies those reacted to all the four DENV serotypes were also screened. For screening, unconjugated peptides and biotinylated DENV NS1 peptides with a biotin–streptavidin system were used to carry out ELISA.

### Development of enzyme-linked immunosorbent assay to screen clones reacting with specific peptide antigens

Initially, hybridoma clones from each fusion were tested against the relevant DENV NS1 peptide using an indirect ELISA. A white 96-well microtiter plate was coated with 200 ng/100 µL of peptide per well in coating buffer (50 mM Tris pH 7.8). Overnight incubation at 4°C was carried out that was followed by washing thrice (3×) with PBST (137 mM NaCl, 2.7 mM KCl, 10 mM Na_2_HPO_4_, 1.8 mM KH_2_PO_4_ with 0.05% Tween 20, pH 7.4). Five percent milk in PBST was added (100 µL/well), and the plate was incubated for 1 h (h) at room temperature (RT). After the washing step (3×) with PBST, 100 µL of hybridoma supernatant diluted 1:1 in 1% milk in PBST was added and was incubated for 2 h at RT. This was followed by washing (3×), then alkaline phosphatase (ALP) conjugated goat anti-mouse IgG (Jackson ImmunoResearch) which I was diluted 1:10,000 in 1% milk in PBST was added and incubated for 1 h at RT. The plate was washed six times (6×) and diflunisal phosphate (DFP) solution which was prepared in substrate buffer (0.1 M NaCl, 1 mM MgCl_2_ in 0.1 M Tris, pH 9.1) was added (100 µL/well). It was incubated for 10 min (min) with gentle shaking and the developing solution (1 M Tris, 0.4 M NaOH, 2 mM TbCl_3_, and 3 mM EDTA) was added (100 µL/well). The solutions were mixed for 10 s, and time-resolved fluorescence was measured using the Wallac 2103 EnVision Multilabel Reader (Perkin Elmer).

### Screening for clones reacting to all four dengue virus serotypes

The above IgG secreting clones were again tested for the secretion of monoclonal antibodies which reacts with all four DENV NS1 peptides. Sheep anti-mouse IgG diluted in coating buffer (sodium carbonate–bicarbonate buffer) was immobilized on a white 96-well microtiter plate (500 ng/well). After a washing step (3×) with PBST, the hybridoma supernatant was diluted (1:1) in 6% bovine serum albumin (BSA) which was added to the plate and incubated for 2 h at RT with gentle shaking. The plate was washed (3×) with washing buffer and 100 µL of biotinylated peptide (50 ng/well) was added followed by incubation for 1 h at RT with gentle shaking. After a washing step (3×), streptavidin–alkaline phosphatase (SA-ALP) (Jackson ImmunoResearch) diluted in 6% BSA (1:1000) was added and incubated for 15 min with gentle shaking. A washing step (3×) was carried out. DFP diluted in substrate solution (1:20) was added and incubated for 10 min with gentle shaking. The developing buffer was added on top, and the plate was allowed to shake gently for 10 s. Time-resolved fluorescence measurement was taken.

### Purification of the antibodies

The hybridoma clones producing the anti-DENV NS1 monoclonal antibodies were selected by limiting dilution. The culture supernatants were further used to obtain purified monoclonal antibodies using affinity chromatography. Selected hybridoma clones were cultured in serum-free media (CD hybridoma medium) (Gibco^®^, United Kingdom) with added l-glutamine (8 mM) for 3–4 weeks, and the culture supernatant was collected for antibody purification. Antibodies present in the culture supernatant were concentrated using ultrafiltration membrane filter units with a nominal molecular weight limit (NMWL) of 10 kD. Purification of monoclonal antibodies was conducted using Protein G Resin according to the instructor manual (GenScript, Technical Manual No. 0209). Dialysis was performed with a molecular porous membrane tubing (molecular cut-off value of 3.5 kDa) (Spectra/Por) using 1 M NaHCO_3_. The purified antibodies were confirmed with DENV-infected patient serum samples by in-house developed sandwich ELISA.

### Generation of dose curve against different amounts of NS1 protein in sample

DENV-positive serum (containing DENV NS1 proteins) was used to coat the micro-well plates by passive adsorption. Patient serum samples diluted (20 μL, 15 μL, 10 μL, and 5 µL) in coating buffer were coated (100 µL/well) on 96-well microtiter plates along with appropriate positive and negative controls. After overnight incubation at RT, the plates were washed (3×) with washing buffer and were blocked with 100 µL of 5% milk in PBST for 1 h at RT with gentle shaking. Following washing (3×), purified monoclonal antibodies diluted in 1% milk in PBST were added (50 ng/well). After incubation for 1 h at RT with gentle shaking, the plates were washed (3×) and goat–anti-mouse IgG HRP (Jackson ImmunoResearch) diluted 1:10,000 in 1% milk in PBST was added and incubated for 1 h at RT with gentle shaking. Subsequently, the plates were washed (6×) and 100 µL of 3,3′,5,5′-tetramethylbenzidine (TMB) solution (BD Biosciences) was added to each well. This was incubated at RT for 15 min and the reaction was terminated by adding 50 µL of 2 M H_2_SO_4_ into each well. The absorbance measurements were taken at 450 nm with a reference filter at 620 nm using the ELISA plate reader (Bio-Rad iMark Microplate Reader).

### Generation of standard curve of anti-DENV NS1 monoclonal antibody

The patient serum samples diluted (1:10) in coating buffer were coated (100 µL/well) on 96-well microtiter plates along with appropriate positive and negative controls. After overnight incubation at RT, the plates were washed thrice with washing buffer and blocked with 100 µL of 5% milk in PBST for 1 h at RT with gentle shaking. Following washing (3×), purified monoclonal antibodies diluted in 1% milk in PBST was added (1:2000, 1:5000, 1:10,000, 1:15,000, 1:20,000, and 1:25,000; v: v). After incubation for 1 h at RT with gentle shaking, the plates were washed (3×) and goat–anti-mouse IgG HRP (Jackson ImmunoResearch) diluted 1:10,000 in 1% milk in PBST was added and incubated for 1 h at RT with gentle shaking. Subsequently, the plates were washed (6×) and 100 µL of TMB solution was added to each well. It was incubated at RT for 15 min and the reaction was terminated by adding 50 µL of 2 M H_2_SO_4_ into each well. The absorbance measurements were taken at 450 nm with a reference filter at 620 nm using the ELISA plate reader.

### Screening of monoclonal antibodies for reactivity with patient serum samples

The purified monoclonal antibodies reacting with all four peptides were tested for reactivity with DENV NS1 native protein using DENV-confirmed patient serum samples. The patient serum samples were confirmed using the SD Bioline Dengue NS1 Ag Detection Kit (SD Bioline, Korea). Patient serum samples diluted (1:10) in coating buffer (sodium carbonate-bicarbonate buffer) were coated (100 µL/well) on 96-well microtiter plates. The same procedure was used as described in the generation of a dose curve against different amounts of NS1 protein in serum samples.

### Screening of monoclonal antibodies for reactivity with patient urine samples

A clear 96-well microtiter plate was coated with 100 µL each sample (diluted 1:4 in coating buffer) along with the negative (coating buffer), sample control (DENV negative urine), and positive (known DENV positive sample) controls. After overnight incubation at RT, the plate was washed 2x with PBS. Blocking was performed for 1 h with 1% skim milk/PBST and it was washed 3x with PBST. DENV NS1 monoclonal antibody was added (500 ng/well) diluted in 1% skim milk/PBST and was incubated for 1 h at RT. The washing step was continued and goat anti-mouse IgG HRP conjugated antibody diluted in 1% skim milk/PBST (1:5000) was added into each well. Incubation was conducted for 2 h at RT and the plate was washed 6x with PBST. TMB solution was added (100 µL/well) and incubated for 20 min at RT. In order to stop the reaction, 50 µL of 2M H_2_SO_4_ was added into each well and absorbance measurements were taken at 450 nm wavelength.

## Results

### Peptides against dengue virus NS1 protein

The amino acid sequences for DENV NS1 protein were analyzed using the Antigen Profiler Peptide Tool by Thermo Scientific and Peptide Property Calculator by GenScript. The protein sequences of the peptides (Masrinoul et al., 2011) were changed according to the sequences of the Sri Lankan isolates (as depicted in red color in Table 1) deposited in NCBI (NCBI accession numbers—DENV1: AEB98757.1, DENV2: ACS32038.1, DENV3: AHG23239.1, and DENV4: AHN50410.1). BioEdit software and NCBI BLAST were used for further analysis of the peptides. According to the analysis, hydrophobicity and percent similarity of the peptides are presented in Table 2. Considering many factors as discussed below, peptides with the following amino acid sequences were designed for the production of anti-DENV NS1 antibodies.

**TABLE 1:** Amino acid sequences of native proteins *vs*. the changes made to the amino acid sequence during peptide designing.

	Original amino acid sequence	New sequence
Peptide 1	PECPDDQRAWNIWEV	PESSDDQRAWNIWEV
Peptide 2	AECPNTNRAWNSLEV	AESPNTNRAWNSLEV
Peptide 3	PECPSASRAWNVWEV	PESPSASRAWNVWEV
Peptide 4	SECPNERRAWNFLEV	SESPNERRAWNSLEV

**TABLE 2:** Percent similarity and hydrophobicity of the designed peptides.

Dengue serotype	Peptide	Similarity (%)			Hydrophobicity
		P1	P2	P3	
1	P1	100			37.57
2	P2	53.3	100		24.76
3	P3	66.6	66.6	100	34.16
4	P4	53.3	80	60	24.87

### Production of anti–dengue virus NS1 monoclonal antibodies

**FIGURE 1:**
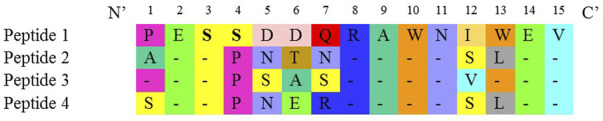
Amino acid sequence of the designed peptides indicating similarity.

Four synthetic peptides designed against the four serotypes of the DENV NS1 region (Figure 1) were used as the immunogen to produce mouse monoclonal antibodies. The fusion of murine splenocytes with murine myeloma cells resulted in a total number of 28 IgG-secreting hybridoma clones out of the 1,830 growing clones. These 28 clones produced antibodies that react with DENV NS1 peptides.

### Screening for clones reacting with all four dengue virus NS1 peptides

**FIGURE 2:**
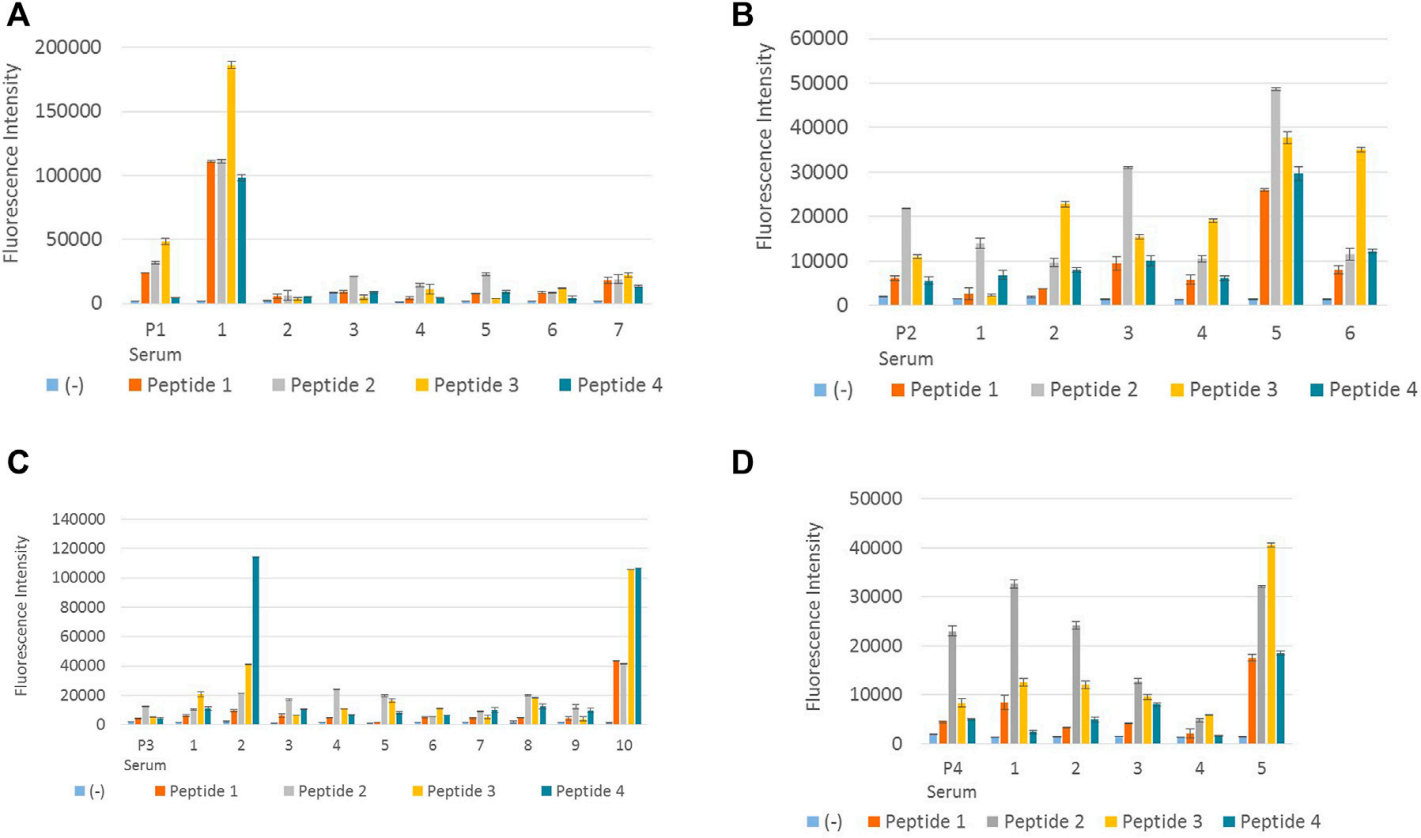
Screening of hybridoma clones reacting with DENV NS1 peptides. **(A)** Fluorescence intensity measurements of IgG-secreting hybridoma clones generated from peptide 1–immunized mouse reacting with all four DENV NS1 peptides. P1 serum denotes polyclonal antiserum from peptide 1–immunized mouse. Columns 1–7 denote supernatant from seven hybridoma clones: P1-18, P1-44, P1-122, P1-195, P1-252, P1-267, and P1-285, respectively. **(B)** Fluorescence intensity measurements of IgG-secreting hybridoma clones generated from peptide 2–immunized mouse, reacting with all four DENV NS1 peptides. P2 serum denotes polyclonal antiserum from peptide 2–immunized mouse. Columns 1–6 denote supernatant from six hybridoma clones: P2-96, P2-256, P2-407, P2-441, P2-679, and P2-731, respectively. **(C)** Fluorescence intensity measurements of IgG-secreting hybridoma clones generated from peptide 3–immunized mouse reacting with all four DENV NS1 peptides. P3 serum denotes polyclonal antiserum from peptide 3–immunized mouse. Columns 1–10 denote supernatant from 10 hybridoma clones: P3-12, P3-28, P3-31, P3-46, P3-70, P3-103, P3-120, P3-123, P3-308, and P3-318, respectively. **(D)** Fluorescence intensity measurements of IgG-secreting hybridoma clones generated from peptide 4–immunized mouse reacting with all four DENV NS1 peptides. P4 serum denotes polyclonal antiserum from peptide 4–immunized mouse. Columns 1–5 denote supernatant from five hybridoma clones: P4-86, P4-104, P4-185, P4-305, and P4-312, respectively. Column (−) in all **(A–D)** denotes the negative control (BSA). The hybridoma supernatant which contains anti-DENV NS1 monoclonal antibodies was diluted 1:1 in 6% BSA. Error bars represent the standard deviation (*n* = 2).

The above 28 clones (Table 3) were tested separately with peptides 1, 2, 3, and 4, with appropriate positive and negative controls, in order to determine the clones which react with all four DENV peptides. Figure 2 illustrates the results obtained from the screening of hybridoma clones reacting with DENV NS1 peptides.

**TABLE 3:** Hybridoma clones producing antibodies reacting with DENV NS1 peptides.

Antigen used	Number of clones	Clones
Peptide 1	7	P1-18, P1-44, P1-122, P1-195, P1-252, P1-267, and P1-285
Peptide 2	6	P2-96, P2-256, P2-407, P2-441, P2-679, and P2-731
Peptide 3	10	P3-12, P3-28, P3-31, P3-46, P3-70, P3-103, P3-120, P3-123, P3-308, and P3-318
Peptide 4	5	P4-86, P4-104, P4-185, P4-305, and P4-312

According to Figure 2A, monoclonal antibody P1-18 resulting from the fusion of DENV 1 peptide–immunized mice showed promising antibody response for all four dengue serotypes. Thus, hybridoma clone P1-18 was eventually expanded in the serum-free media and purified using protein G columns. Purified anti-DENV NS1 monoclonal antibodies (P1-18) were used to confirm the antibody reactivity with DENV NS1 native protein using DENV-confirmed serum and urine samples.

**FIGURE 3:**
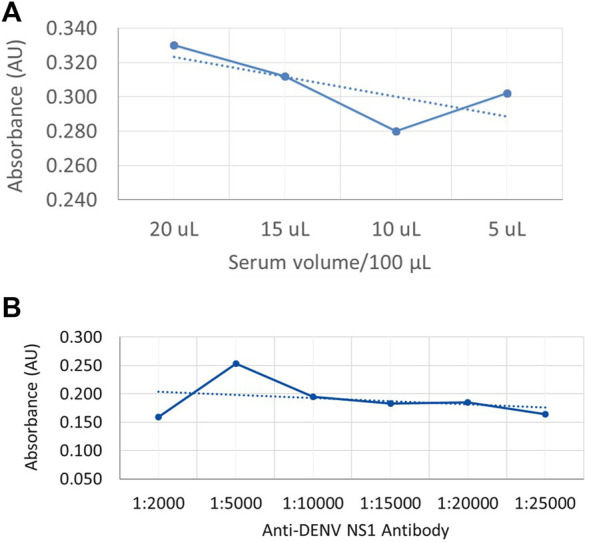
Dose-dependent indirect ELISA curve for different amounts of NS1 protein in the sample and a standard curve for anti-DENV NS1 antibodies **(A)** Absorbance values against DENV-positive serum volume, with NS1 protein as coating antigen. **(B)** Absorbance values against known anti-DENV NS1 monoclonal antibody concentration as the capture antibody.

In order to generate a dose curve against different amounts of NS1 protein in a sample, DENV-positive serum (DENV NS1 proteins) was used to coat the microwell plates by passive adsorption, and a dilution series was used to generate a standard curve of anti-DENV NS1 monoclonal antibody (P1-18) as shown in Figures 3A,B, respectively.

**FIGURE 4:**
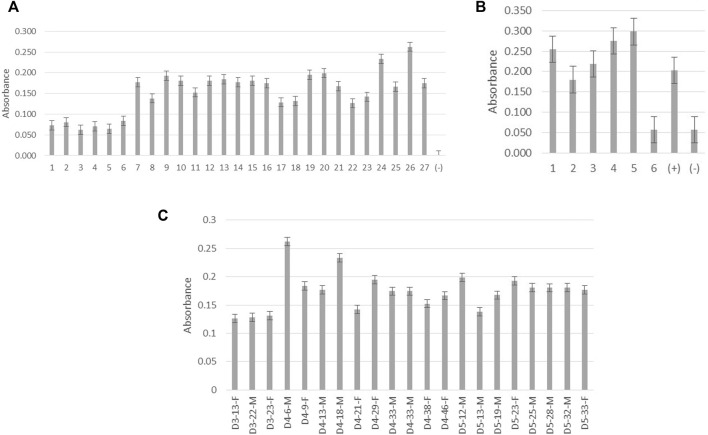
Screening of monoclonal antibody P1-18 for reactivity with native DENV NS1 protein. **(A)** Absorbance measurements of P1-18 antibody reacting with patient serum samples. Columns 1–6 denote samples from dengue-negative patients with febrile illnesses. Columns 7–27 denote samples from dengue-positive patients. Column (−) represents the blank **(B)** Absorbance measurements of P1-18 antibody reacting with patient urine samples. Columns 1–5 denote samples from dengue-positive patients. Column 6 denotes a sample from dengue-negative/healthy individuals. Columns (+) represent dengue-positive serum samples and column (−) denotes the blank. **(C)** Absorbance measurements of P1-18 antibody reacting with patient serum samples against days of fever (DOF), age (in years), and gender (female: F and male: M). All the absorbance measurements were taken at 450 nm wavelength. D3 represents the third day from the onset of fever, D4 represents the fourth day from the onset of fever, and D5 represents the fifth day from the onset of fever. Error bars represent the standard deviation (*n* = 3).

Antibodies from the clone P1-18 were further verified using previously confirmed dengue-positive and dengue-negative patient serum and urine samples as illustrated in Figure 4A,B. High reactivity can be observed for both serum and urine samples obtained from dengue-positive patients.

The development of monoclonal antibodies against dengue NS1 full-length protein and recombinant proteins has been carried out in various studies. Nevertheless, utilizing peptides in monoclonal antibody production have not been examined comprehensively. Thus, in this study, monoclonal antibodies were produced using DENV NS1 synthetic peptides, which can capture all four serotypes of the dengue virus. Synthetic peptides provide a cost-effective method rather than using the whole viral protein to produce monoclonal antibodies which ultimately affected the duration of monoclonal antibody production. These antibodies can be used for further development of immunoassays and rapid diagnostics for the detection of dengue infection.

## Discussion

Oxidation causes major degradation in peptides, both in solution and lyophilized form. There are many amino acids such as methionine (Met), cysteine (Cys), histidine (His), tryptophan (Trp), and tyrosine (Tyr) that may undergo oxidation. In therapeutic proteins, Met and Cys are the most common residues that encounter oxidation reactions during normal storage conditions (Tabrizi et al., 2012). Cys is easily oxidized to yield cysteine disulfide and may be oxidized to intra-chain disulfide linkages which can ultimately lead to protein aggregation as cited by Wiedemann et al. (2020). Some oxidation reactions such as oxidation of Met to methionine sulfoxide are not involved in the resulting huge conformational changes those ultimately affect protein antigenicity and thereby the immunogenicity of the peptides. Nevertheless, the oxidation of Cys residues can result in disulfide bridges which can create huge changes in antigenicity and solubility (Wiedemann et al., 2020). Due to significant conformational changes that can occur, the replacement of Cys with serine (Ser) is an ideal pathway to prevent such oxidation reactions. Ser is a stable amino acid with a hydroxyl group, and it simulates the size and polarity of Cys. Furthermore, Ser mutant often preserves the full biological activity (Banga, 2015). Therefore, Cys residues were replaced with Ser. The changes are colored in green, as depicted in Table 1.

Trp can also affect the purity of the peptide, as a result of its chances of oxidation during synthesis. In these DENV NS1 peptides, Trp is not avoided since it can affect antigenicity. To evade the consequences, N-terminal Cys was added for carrier protein conjugation.

During the peptide manufacturing process, each coupling step (that is, each amino acid addition) was followed by a capping step, which terminates any incomplete peptide chains (Howl, 2005). Since peptides are made from right to left, if a Cys is added to the N-terminus, only full-length peptides will have an N-terminal Cys that can be conjugated to the carrier protein. This step is carried out in order to ensure that there are never any deletions in peptides, in the final preparations, only truncations can occur. So when using N-terminal Cys, peptide purity is less critical since only full-length peptides will be conjugated and the shorter peptides are removed during dialysis (Stawikowski and Fields, 2012).

Solubility is a key factor for an antigen to be effective. Peptides with a high number of hydrophobic amino acids such as Ile, Leu, Met, Phe, Trp, and Val will be most likely insoluble in aqueous solutions due to their non-polarity (Trevino et al., 2007). In general, peptide solubility in aqueous solutions is highly affected by the hydrophobicity of the peptide. When hydrophobic peptides carry 50% or more hydrophobic amino acid residues, it is most probably insoluble or moderately soluble in aqueous solutions. In designing soluble peptides, one out of every five amino acids should be a charged amino acid. In order to have a soluble peptide, hydrophilic peptides should contain more than 25% of charged residues and less than 25% of hydrophobic amino acids. Thus, the number of hydrophobic residues in the peptides was maintained below 50%, ensuring one in every five amino acids was charged (Merrifield, 1963; Ji and Feng, 2008; Lloyd-Williams et al., 2020). Therefore, from the original amino acid sequence of the DENV NS1 protein, the amino acid regions which had charged amino acids were selected, in order to have a net (negative or positive) charge in the full-length peptide. As an antigen, the optimal peptide length for binding with an antibody is considered to be between 8 and 12 amino acids. In general, it is believed that the central 5–7 residues contribute to the majority of the specific contacts of the antigen. Nevertheless, currently, peptide antigens with 20 amino acid residues are most commonly used (Hancock and OReilly, 2005; Lloyd-Williams et al., 2020). Considering the length, lengthy peptides can be highly immunogenic, but at the same time, they can create higher chances for cross-reactivity. A short peptide can improve the specificity as an antigen, but it can also negatively affect immunogenicity. In order to obtain both highly conserved and variable regions among the four DENV serotypes, and considering all the previously discussed factors, a peptide length of 15 amino acids was selected. The hydrophobicity of the peptides was calculated using the ThermoFisher peptide analyzing tool.

Using peptides as antigens requires the conjugation of a carrier protein of interest. This is important since the peptides are small molecules that alone do not tend to be immunogenic, thus possibly eliciting a weak immune response. The carrier protein contains many epitopes that stimulate T-helper cells, which help induce the B-cell response. The most commonly used carrier protein is KLH, due to its higher immunogenicity (Goding, 1996; Swaminathan et al., 2014). Furthermore, another strength of selecting KLH over BSA is that BSA is used as a blocking buffer in many experimental assays, and since the antisera raised against the peptides is conjugated to BSA, it will also contain antibodies to BSA and false-positives may result. Therefore, for the peptides used in this study, KLH was selected as the carrier protein. Some other factors were also deliberated in the designing of peptides. Multiple (more than three) serine or proline and glutamine residues in the sequences were avoided as a result of significant deletions that can occur during synthesis, as well as to minimize hydrogen bonding between peptides. Proline residues can undergo cis/trans isomerization and reduce peptide purity (Sarkar et al., 2007). Finally, N-terminal Cys was added to the peptide sequence to allow peptide conjugation to a carrier protein (Banga, 2015).

During the immunization of mice for antibody production, these peptides elicit an immune response that targets the epitopes given by the peptides. Therefore, it can control the antigen-binding sites of the monoclonal antibodies produced. In this study, the designed DENV NS1 peptides were supposed to have serotype-specific epitopes and common epitopes due to the variable and conserved amino acid sequences available in the selected region, respectively.

According to previously published articles, six amino acids have been considered to be the minimum number of amino acids that get together to make an epitope (Hebbes et al., 1989). Compared to the amino acid sequences of the designed peptides, the antibodies produced by clone number P1-18 might have targeted an epitope near the C-terminal of the peptide antigen. The reason why clone P1-18 reacts with peptide 3 with a considerable high efficacy than it does with peptide 1 can be because of the similarity between peptides 1 and 3 at their the C-terminal end with only one amino acid variation. The amino acid isoleucine is used in P1, whereas valine is used in P3. These two amino acids are branched-chain amino acids. As a consequence, their structural configuration can simulate similarity in antigenicity. Clone P2-731 which is obtained from fusion 2, produces antibodies with higher reactivity for peptide 3, which may also occur as a result of the similarities in the conserved region of peptides 2 and 3. Fusion 4 has also produced clones reacting satisfactorily with peptide 2, instead of its immunized peptide (P4). This homogeneity can also be a result of the conserved region at the C-terminal of peptides 2 and 4.

The serum samples used in this study were primarily confirmed using a commercially available dengue detection kit. For the initial confirmation, the SD Bioline Dengue NS1 Ag Detection Kits were utilized. This is a one-step immunochromatographic assay designed to detect DENV NS1 antigen to determine the presence of dengue virus in the human whole blood, serum, and plasma. According to the SD Bioline manufacturer’s instructions, in order to detect dengue NS1 antigen in clinical samples, 100 μL of the test sample should be added to the sample well of the kit, and the results can be read at 15–20 min. According to studies, the sensitivity and specificity of the SD Bioline Dengue Duo NS1 Ag kit were 48.62% and 100%, respectively (Jang et al., 2019).

According to the available literature, the secreted form of DENV NS1 protein is available in DENV patients from 1 to 10 μg/ml depending on several factors (Alcon-LePoder et al., 2005). Therefore, the coating volumes for the generation of the dose curve were selected as 5 μL, 10 μL, 15 μL, and 20 µL. According to Figure 3A, the absorbance values show a reduction with the reduction of serum volume. However, the absorbance value is increased in 5-µL coated wells which may be due to less competition from the other serum proteins that are bound on the plate. According to Figure 3B, the highest absorbance value was offered when the antibody volume used was 1:5000 (v/v) in 1% skim milk in PBST as the capture antibody, where the initial concentration of the purified anti-DENV NS1 monoclonal antibody was 1 mg/ml.

Varying concentrations of NS1 protein could be present in patients according to the patient’s level of immunity and also the severity of the virus despite days of fever, gender, and patient’s age as is evident from Figure 4C. According to studies conducted by Korhonen et al. (2014), the concentration of the DENV NS1 protein detected in the serum is significantly high in patients with dengue fever when compared with those who had dengue hemorrhagic fever and dengue shock syndrome. The concentration of the secreted dengue NS1 protein in urine is believed to be high in patients with severe or complicated dengue infection (Chuansumrit et al., 2011). As illustrated in Figure 4B, anti-DENV NS1 monoclonal antibody produced in this study reacts excellently with dengue-positive urine samples, and this provides a high potential to use this antibody in the development of diagnostics targeting patient urine samples.

## Conclusion and recommendations

During the study, we successfully produced anti-DENV NS1 monoclonal antibodies using synthetic peptides for the detection of DENV infection. Peptide antigens are highly valuable in the production of monoclonal antibodies, which is extremely economical and easy for laboratory experiments. Finally, the findings of the current research can be used in the early detection of DENV. Collecting urine samples for the detection of dengue infection is noninvasive and can be done by untrained individuals, making it a convenient way for sample collection. Using these monoclonal antibodies which can detect DENV NS1 protein in both the patient’s blood and urine at an early stage of infection can finally result in the prevention of dengue epidemics through early diagnosis, treatment, and patient management in order to safeguard human and economic wellness.

## Data Availability

The data sets presented in this study can be found in online repositories. The names of the repository/repositories and accession number(s) are https://www.ncbi.nlm.nih.gov/, AEB98757.1; https://www.ncbi.nlm.nih.gov/, ACS32038.1; https://www.ncbi.nlm.nih.gov/, AHG23239.1; https://www.ncbi.nlm.nih.gov/, AHN50410.1.
